# Targeting the Bcl-2 Family in B Cell Lymphoma

**DOI:** 10.3389/fonc.2018.00636

**Published:** 2019-01-08

**Authors:** Clare M. Adams, Sean Clark-Garvey, Pierluigi Porcu, Christine M. Eischen

**Affiliations:** ^1^Department of Cancer Biology, Sidney Kimmel Cancer Center, Thomas Jefferson University, Philadelphia, PA, United States; ^2^Internal Medicine Residency Program, Department of Internal Medicine, Sidney Kimmel Medical College, Thomas Jefferson University, Philadelphia, PA, United States; ^3^Division of Hematologic Malignancies and Hematopoietic Stem Cell Transplantation, Department of Medical Oncology, Sidney Kimmel Cancer Center, Thomas Jefferson University, Philadelphia, PA, United States

**Keywords:** BCL-2, BCL-W, B cell lymphoma, apoptosis, venetoclax, navitoclax, BH-3 mimetic, CLL

## Abstract

Although lymphoma is a very heterogeneous group of biologically complex malignancies, tumor cells across all B cell lymphoma subtypes share a set of underlying traits that promote the development and sustain malignant B cells. One of these traits, the ability to evade apoptosis, is essential for lymphoma development. Alterations in the Bcl-2 family of proteins, the key regulators of apoptosis, is a hallmark of B cell lymphoma. Significant efforts have been made over the last 30 years to advance knowledge of the biology, molecular mechanisms, and therapeutic potential of targeting Bcl-2 family members. In this review, we will highlight the complexities of the Bcl-2 family, including our recent discovery of overexpression of the anti-apoptotic Bcl-2 family member Bcl-w in lymphomas, and describe recent advances in the field that include the development of inhibitors of anti-apoptotic Bcl-2 family members for the treatment of B cell lymphomas and their performance in clinical trials.

## Introduction

Acquiring resistance to apoptosis, a highly-regulated, evolutionarily conserved process, is a characteristic shared among all types of cancer, including B cell lymphomas (1). For a cell to divide and grow uncontrollably, as malignant cells do, it must not only hijack the normal cellular growth pathways, but it must also avoid cellular death signals. During lymphomagenesis, B cells encounter a broad range of stress stimuli, including oncogene activation, DNA damage, and oxygen and cytokine deprivation, all of which can elicit an apoptotic cell death response. Apoptosis is regulated by complex interactions between pro-apoptotic and anti-apoptotic members of the B cell lymphoma-2 (Bcl-2) protein family (2). Thus, a delicate balance between members of the Bcl-2 family dictate whether the B cell will live or die under stress conditions. As such, alterations that deregulate the apoptotic process lead to increased survival and facilitate lymphomagenesis (3). Moreover, these alterations can render lymphoma cells refractory to therapies that are designed to induce death (4). Specifically, overexpression of the anti-apoptotic Bcl-2 family members and/or reduced expression of specific pro-apoptotic members are a common feature shared among B cell lymphomas (4, 5). Much of what we know today about the Bcl-2 family and its role in B cells and B cell lymphoma comes from decades of research utilizing genetically engineered mice and a mouse model of Myc oncogene-induced B cell lymphoma [Eμ-*myc* transgenic mice, (6)]. However, recent discoveries and low complete response rates in clinical trials with targeted therapy against BCL-2 in lymphoma reveal significant gaps in knowledge remain (7–9). This review comprehensively examines each member of the Bcl-2 protein family, defining their contribution to B cell lymphomagenesis through mouse models and the alterations that occur in them in human B cell lymphomas, including our recent discovery of Bcl-w overexpression. In addition, this review also describes current therapeutic efforts to target specific anti-apoptotic Bcl-2 family members in lymphoma patients alone or in combinations to improve survival.

## Bcl-2 Protein Family and apoptosis

**Figure 1:**
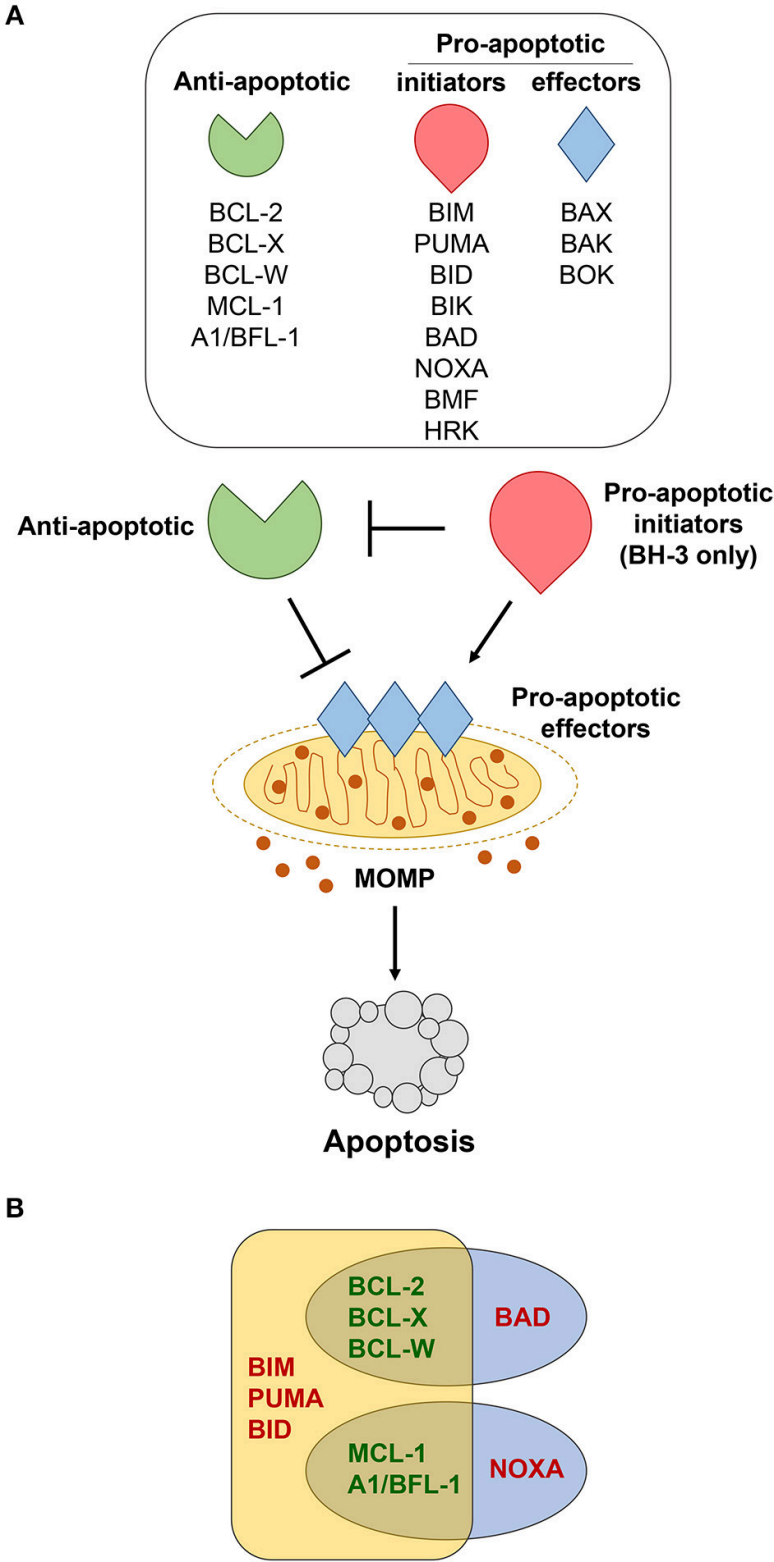
Bcl-2 family members regulate apoptosis. **(A)** Various cellular stressors induce apoptosis through the intrinsic, mitochondrial pathway, which is regulated by the Bcl-2 family of proteins. These stress signals activate pro-apoptotic BH-3 only initiators (red), which inhibit the anti-apoptotic proteins (green). This, in turn, allows the pro-apoptotic effectors (blue) to be activated. Activation of the effector proteins results in their oligomerization and subsequent mitochondrial outer membrane permeabilization (MOMP), enabling the release of apoptotic factors that initiate the caspase cascade and final stages of cellular destruction. **(B)** Pro-apoptotic BH-3 only proteins bind to anti-apoptotic Bcl-2 family members with different affinities. BIM, PUMA, and BID bind strongly to all anti-apoptotic Bcl-2 proteins, whereas BAD binds preferentially to BCL-2, BCL-X, and BCL-W, and NOXA binds preferentially to MCL-1 and A1/BFL-1.

B cells continuously monitor their environment and make decisions as to whether they should live or die. The Bcl-2 protein family are the central gatekeepers of the intrinsic or mitochondrial apoptotic response. The family is comprised of structurally-related proteins with opposing functions that either promote or inhibit apoptosis by interacting with one another (10). The Bcl-2 family is typically classified into three groups, including pro-apoptotic initiators, pro-apoptotic effectors, and anti-apoptotic proteins (Figure 1A). The apoptotic-promoting effects from the pro-apoptotic initiators and effectors are countered by their direct interaction with the anti-apoptotic family members. It is this delicate and dynamic balance between the pro- and anti-apoptotic Bcl-2 family members that governs whether a B cell undergoes apoptosis or survives. We discuss the consequences of alterations for each of the Bcl-2 family members in lymphoma in mouse models and make comparisons to what is observed in human lymphomas (see Table 1).

**Table 1:** Alterations in Bcl-2 family members in mouse models and human lymphoma.

**Family member**	**Mouse models**	**Human patients**
**PRO-APOPTOTIC**		
BIM	Loss accelerates Myc-driven BCL (11)	Deleted in 20% MCL (12); SNPs present in FL, DLBCL, CLL (13); Low mRNA expression in 40% BL (14)
PUMA	Loss accelerates Myc-driven BCL (15, 16)	Low mRNA expression in 40% BL (15)
NOXA	Loss does not accelerate Myc-driven BCL, but does increase B cell numbers (16)	Unknown
BAD	Loss accelerates Myc-driven BCL (17); 25% with deletion develop DLBCL at old age (18)	No known link with DLBCL
BID	Loss causes CMML (19)	Unknown
BIK	Loss does not accelerate Myc-driven BCL (20) and has no effect on hematopoietic cells (21)	Somatic missense mutations in FL, MZL, and DLBCL (22)
BMF	Loss accelerates Myc-driven BCL and increases B cell numbers (17)	Reduced protein levels in BL (17)
BAK	Null mice are phenotypically normal (23); Unknown effects on Myc-driven BCL	Unknown
BAX	Null mice have mild lymphoid hyperplasia (24); Loss accelerates Myc-driven BCL (25)	Unknown
BOK	Loss does not accelerate Myc-driven BCL (26)	Unknown
**ANTI-APOPTOTIC**		
BCL-2	Null mice have a premature death (27); Overexpression increases B cells and accelerates Myc-driven BCL (28)	Translocated in 90% FL (29) and 20% DLBCL (30); Somatic mutations in FL associated with transformation and reduced survival (31); Increased mRNA levels linked to reduced survival (31); Increased mRNA in a subset of MZL (32) and protein in MCL (33)
BCL-X	Null mice are embryonic lethal (34, 35); Loss delays Myc-driven BCL (36); Overexpression increases mature lymphocytes (37); overexpression with Myc causes lymphoproliferation and plasma cell malignancy (38)	Overexpressed in subset of BL (9), FL (9, 39), DLBCL (9, 39), and MCL (9, 40); Low protein expression in MZL (33); Increased mRNA in MZL (9); High mRNA and protein expression in MM (41–44)
MCL-1	Null mice are embryonic lethal (45–47); Loss delays Myc-driven BCL (48, 49); Overexpression increases B cells (50, 51) and accelerates Myc-driven BCL (52)	Amplification or chromosomal gains in 20–25% ABC DLBCL (53); Increased mRNA in CLL (54, 55) and MM (56) and correlated with disease progression in MM (57); Low protien levels in MCL (33)
A1/BFL-1	Null mice are embryonic lethal (58, 59); Overexpression does not accelerate Myc-driven BCL (60)	No change (9) or elevated mRNA in DLBCL (61); Elevated mRNA in CLL (62, 63); Low mRNA levels in BL (9)
BCL-W	Null male mice are sterile (64, 65); Loss delays Myc-driven BCL (8); Overexpression accelerates Myc-driven leukemogenesis (66)	Overexpressed in BL, DLBCL, FL, MZL, and MCL (8, 9)

*BCL, B cell lymphoma; MCL, mantle cell lymphoma; SNP, single nucleotide polymorphism; FL, follicular lymphoma; DLBCL, diffuse large B cell lymphoma; CLL, chronic lymphocytic leukemia; BL, Burkitt lymphoma; CMML, chronic myelomonocytic leukemia; MZL, marginal zone lymphoma; MM, multiple myeloma; ABC, activated B cell subtype of DLBCL*.

### Pro-apoptotic Bcl-2 Family Members

Members of the Bcl-2 protein family share sequence homology within conserved regions known as Bcl-2 homology (BH) domains, which dictate structure and function (67, 68). All anti-apoptotic family members and a subset of pro-apoptotic members are multi-domain proteins, sharing sequence homology within three to four BH domains. A subset of pro-apoptotic Bcl-2 family members known as BH-3 only proteins only contain the BH-3 domain, which is known as the minimal death domain that is required for binding the multi-domain Bcl-2 family members (69).

#### BH-3 Only Proteins: Initiators of Apoptosis

The BH-3 only group of pro-apoptotic Bcl-2 proteins consists of Bim (BCL2L11), Puma/BBC3, Bad (Bcl-2/Bcl-x-associated death promoter), Bid (BH-3 interacting-domain death agonist), Bik (Bcl-2-interacting killer), Noxa/PMAIP1, Bmf (Bcl-2-modifying factor), and Hrk (Harakiri) [Figure 1A, (70)] and are essential for initiating the apoptotic cascade. While the BH-3 only proteins can initiate apoptotic signaling by binding directly to the anti-apoptotic Bcl-2 proteins, thereby freeing up Bax and Bak to undergo homo-dimerization, some have been reported to bind directly to and activate Bax and Bak (71). Years of studies using mouse models have revealed certain BH-3 only proteins are preferentially solicited in response to different apoptotic stimuli (72–76).

BH-3 only proteins serve as the first responders to cellular insults, including from dysregulation of oncogenes, such as Myc, and serve as a blockade against the development of B cell lymphoma. For example, loss of Bim or Puma accelerated Myc-driven B cell lymphoma development in a mouse model engineered to overexpress Myc in B cells (Eμ-*myc* transgenic) (11, 15, 16). Loss of BIM may also contribute to human lymphomas, as ~20% of mantle cell lymphomas (MCL) have deleted both alleles of *BIM* (12). In addition, single nucleotide polymorphisms in the *BIM* gene have been associated with risk of developing follicular lymphoma (FL), diffuse large B cell lymphoma (DLBCL), and chronic lymphocytic leukemia (CLL) (13). Furthermore, ~40% of human Burkitt lymphomas express very low levels of *BIM* or *PUMA* mRNA, which may be the result of epigenetic silencing (14, 15). In contrast to Bim and Puma, loss of Noxa had no effect on Myc-induced B cell lymphomagenesis in mice, but did increase the number of B lineage cells (16). It is unknown whether NOXA loss contributes to human B cell lymphoma. A quarter of mice with deletion of *Bad* develop DLBCL at old age, suggesting that it may have a tumor suppressive function in mature B cells (18). Deletion of *Bad* also accelerated Myc-induced B cell lymphoma (17). Despite the findings in mice, BAD loss has not been linked to DLBCL in humans. Deletion of *Bid* did not result in B cell lymphoma development in mice. Instead, chronic myelomonocytic leukemia emerged in *Bid*-null mice after a long latency period, indicating Bid function is critical for the myeloid lineage (19). It is unknown if alterations in BID contribute to human lymphoma. In mice, loss of *Bik* alone had no effect on hematopoietic cells and did not accelerate Myc-induced B cell lymphoma development, suggesting that it has no role in B cells (20, 21). However, somatic missense mutations have been observed in *BIK* in B cell lymphomas in humans, including FL, marginal zone (MZL), and DLBCL (22), suggesting that its loss may contribute to these lymphomas. Loss of Bmf in mice increased B cell numbers and cooperated with Myc overexpression to accelerate lymphomagenesis; preferentially developing an IgM+ B cell lymphoma (17). Reduced levels of BMF were observed in Burkitt lymphoma patient samples and cell lines (17). Together, the data indicate that each pro-apoptotic BH-3 only family member may facilitate tumor suppression in specific hematopoietic cells, but not all may have a role in B cells.

#### Bax, Bak, and Bok: Effectors of Apoptosis

Activation of Bax and Bak, and possibly the lesser-studied Bok, involves homo-dimerization followed by oligomerization within the outer mitochondrial membrane (71). This conformational change induces mitochondrial outer membrane permeabilization (MOMP) by creating a pore and triggering the release of apoptosis-inducing proteins, such as cytochrome *c* and second mitochondria-derived activator of caspase (Smac)/direct IAP-binding protein with low pI (DIABLO) from the mitochondria [Figure 1A, (77)]. Following this release, the caspase cascade is activated, ultimately resulting in the proteolysis of intra-cellular proteins and cellular destruction.

The combined function of Bax and Bak is critical for development and mediating apoptosis. Mice lacking both Bak and Bax have severe developmental defects and have hematopoietic cells that are resistant to diverse stimuli that activate the intrinsic apoptotic pathway (23, 78). Mice lacking either Bak or Bax are phenotypically normal (23) or have mild lymphoid hyperplasia, respectively (24). Using the Eμ-*myc* mouse model of Myc-driven B cell lymphomagenesis, loss of Bax accelerated lymphoma development (25), but the effects of inactivation of Bak in Myc-induced B cell lymphoma have not been reported. Loss of Bok did not alter Myc-induced B cell lymphoma development (26). In humans, mutations in *BAX* that caused a frameshift were detected in cell lines derived from hematologic malignancies that did not include lymphoma and were associated with resistance to cell death and microsatellite instability (79–81). Alterations of BAX, BAK, or BOK have not been reported in human lymphoma; thus, loss/inactivation of BAX, BAK, or BOK either does not occur or is a rare event in human B cell lymphoma.

### Anti-apoptotic Bcl-2 Family Members

Acquired resistance to apoptosis is regarded as one of the hallmarks of cancer (1). Accordingly, evidence continues to reveal that elevated expression of anti-apoptotic Bcl-2 family members (Bcl-2, Bcl-x, Bcl-w, Mcl-1, A1/Bfl-1) is one of the major contributing factors to B cell lymphomagenesis (2). Distinct biological roles for the individual anti-apoptotic Bcl-2 family members have been unveiled by genetically-engineered mouse models (3). Additionally, it has been hypothesized that levels of expression of individual anti-apoptotic Bcl-2 family proteins may be an indication of how dependent a cell is on the protein to maintain its survival (82). The mechanism by which the anti-apoptotic Bcl-2 family proteins inhibit apoptosis is predominately governed by their capacity to bind and sequester the pro-apoptotic BH-3-only proteins or Bax and Bak, ultimately preventing mitochondrial membrane permeabilization [Figure 1A, (83)]. The ability of the anti-apoptotic Bcl-2 proteins to bind pro-apoptotic Bcl-2 proteins does vary and depends, at least in part, on the apoptotic stimuli and which BH-3 only proteins are expressed and/or activated [Figure 1B, (10, 84, 85)]. We will discuss each of the anti-apoptotic Bcl-2 family members, focusing particular attention on Bcl-w to highlight its newly-exposed contributions to B cell survival and lymphomagenesis.

#### BCL-2

*BCL-2* is translocated *t*(14;18)(q32;q21) to the immunoglobulin heavy chains, resulting in its constitutive expression in 90% of FL (29, 86–90). *Bcl-2* knockout mice showed that Bcl-2 is required for normal B cells to survive (27), providing evidence for why B cell lymphomas would select for its overexpression. Somatic mutations in *BCL-2* in FL are often associated with transformation of this indolent disease to more aggressive diffuse large B cell lymphoma (DLBCL) and decreased patient survival (31). Some of these mutations increased the affinity of BCL-2 to pro-apoptotic BH-3 only proteins and have also been detected in lymphoid cell lines (91, 92).

Approximately 20% of *de novo* DLBCL also harbor *BCL-2* translocations (30). Increased *BCL-2* expression has been linked to reduced survival of patients with DLBCL (93, 94). The first large-scale gene expression profiling studies classified DLBCL into two major molecular subgroups, germinal center B cell (GCB) and activated B cell (ABC) subtypes (95). GCB DLBCL that have increased levels of BCL-2 is most often due to a *t*(14;18) translocation (96), whereas in ABC DLBCL, amplification of the *BCL-2* gene is more often observed (93, 97). Using gene expression profiling, Iqbal and colleagues reported a significant correlation between elevated *BCL-2* mRNA expression and poor overall survival within the ABC subtype (93). We also observed elevated levels of *BCL-2* in both subtypes of DLBCL, but *BCL-2* was more highly expressed in the ABC subtype than the GCB subtype (9). More recently, new classifications of DLBCL subtypes have been reported by two groups (98, 99). Shipp and colleagues described a new classification of DLBCL subtypes based on genetic signatures of low-frequency alterations, recurrent mutations, somatic copy number alterations, and structural variants. One of their two distinct subtypes of GCB-DLBCL (cluster 3) has structural variants of BCL-2 and correlate to poor risk (98). Their ABC-DLBCL subtype (cluster 5) had amplification of *BCL-2* (98), as previously described (93). Staudt and colleagues also reported new classifications of DLBCL that include four new genetic subtypes, one of which is EZB, which has EZH2 mutations and BCL-2 translocations (99).

DLBCL lymphomas that contain both rearrangements in *BCL-2* and translocation of *MYC* are classified as “double hit lymphomas” (DHL) and represent ~10% of DLBCL cases (100, 101). DLBCLs that co-express high levels of MYC and BCL-2 proteins due to mechanisms other than chromosomal translocations are referred to as “dual expresser lymphomas” (DEL) and represent ~30% of DLBCL (102, 103). Both DHL and DEL tend to be clinically more aggressive and have a higher frequency of treatment failure than those that are non-DHL or non-DEL (102, 103). Because of this, these subtypes of DLBCL have become a new biomarker-defined subset, which illustrates the importance of knowing the status of MYC and BCL-2 to help guide treatment and monitoring of patients. These results also provide impetus to investigate the expression of other anti-apoptotic BCL-2 family members in lymphomas, as others may also alter prognosis.

Besides *BCL-2* translocation and amplification, additional mechanisms are reported to contribute to its increased expression, including *BCL-2* gene rearrangement (104), promoter hypomethylation (105), promoter hypermutation (106), and phosphorylation (107). A small subset of mantle cell lymphoma (MCL) have increased expression of *BCL-2* (33). Additionally, BCL-2 can also become overexpressed in the indolent MZL (32). For patient samples of both MCL and MZL, BCL-2 overexpression is reportedly caused by non-genomic changes to BCL-2 (33, 108). *BCL*-*2* expression can also be regulated by non-coding RNA. In B cells, *BCL-2* expression is negatively regulated by the miR-15a/miR-16-1 cluster (109, 110). This region is deleted or inactivated by mutations in ~70% of CLL (110); however, no other B cell malignancy has been associated with loss of the region (111, 112).

Although the belief in the importance of BCL-2 in human B cell lymphomas is firmly embedded, two different transgenic mice generated ~30 years ago revealed that Bcl-2 is not a driver of B cell lymphoma, but increased levels in B cells did lead to their accumulation (113, 114). Bcl-2 overexpression did cooperate with Myc overexpression to accelerate B cell lymphomagenesis (28). The requirements of BCL-2 in the continued survival of human B cell lymphomas is just now being explored with some surprising results, as described below.

#### BCL-X

Shortly following the cloning of the *BCL-2* gene, the gene coding for *BCL-X* (BCL2L1 for Bcl-2 like 1) was discovered due to its high level of sequence similarity to *BCL-2* (115, 116). Similar to *Bcl-2* transgenic mice, mice engineered to overexpress Bcl-x renders lymphoid cells resistant to numerous apoptotic stimuli and causes an abnormal accumulation of mature lymphocytes, but not overt lymphoma development (37). However, mice double-transgenic for *Bcl-x* and *Myc* in B cells developed lymphoproliferative disease and plasma cell malignancies (38). Knocking out *Bcl-x* revealed that it is not required for lymphocyte development, but is critical for erythropoiesis and platelets (34, 35, 117, 118). Loss of Bcl-x did delay Myc-induced B cell lymphoma development, suggesting that under conditions of oncogenic stress, B cells may rely on Bcl-x for survival (36).

Burkitt lymphomas can select to overexpress *BCL-X* (9). Elevated *BCL-X* expression has been detected in other B cell non-Hodgkin lymphomas, including FL and DLBCL, as well as T cell non-Hodgkin lymphomas (39). Moreover, it has been demonstrated that in DLBCL, elevated expression of *BCL-X* mRNA is associated with a chemoresistant, short-lived group of patients (119). Like BCL-2, selection for BCL-X overexpression occurs in a subset of MCL (40). Moderate levels of BCL-X protein were reported in several cases of CLL, FL, and MCL, but it was lowly expressed in MZL (33). In a large-scale analysis of gene expression profiling data, we reported that *BCL-X* mRNA was significantly elevated compared to normal human B cells in multiple types of non-Hodgkin B cell lymphoma, including Burkitt, DLBCL, FL, MCL, and MZL (9). Unlike BCL-2, which is lowly expressed in multiple myeloma (MM), levels of BCL-X are much higher and may therefore be a critical survival factor for MM (41–44). To date, no chromosomal translocation involving *BCL-X* has been reported in human tumor samples, but somatic copy number amplifications have been detected in hematopoietic malignancies, including non-Hodgkin lymphomas (120). High levels of *BCL-X* have also been attributed to the loss or silencing of the let-7 family of miRNA that targets *BCL-X* (109, 121, 122). Therefore, BCL-X likely contributes to B cell lymphomas.

#### MCL-1

Upregulation of the anti-apoptotic Bcl-2 family member Mcl-1 likely also contributes to lymphomagenesis. Mcl-1 was first identified in an immortalized myeloid leukemia-derived cell line, and consequently named myeloid cell leukemia 1 (123). Consistent with Bcl-2 and Bcl-x overexpression models, Mcl-1 overexpression in transgenic mice renders hematopoietic cells largely resistant to varying apoptotic stimuli and causes the accumulation of mature B and T lymphocytes (50, 51). In addition, half of the *Mcl-1* transgenic mice develop B cell lymphomas within two years (52). *Mcl-1* knockout mice are embryonic lethal, but conditional knockout of *Mcl-1* in mice shows a requirement for Mcl-1 in hematopoietic stem cell and neutrophil survival (45–47). Furthermore, loss of Mcl-1 inhibited Myc-induced B cell lymphomagenesis in mice (48, 49).

A somatically acquired increase in *MCL-1* copy number has been documented in a variety of non-hematopoietic malignancies, but only in a limited number of non-Hodgkin lymphomas (120). Gene amplification or chromosomal gains of *MCL-1* occur in 20–25% of the activated B cell (ABC) subtype of DLBCL (53). Although individual lymphomas may select for overexpression of *MCL-1*, we determined *MCL-1* expression was not elevated in a cohort of Burkitt lymphomas compared to normal human B cells, and that it was also not significantly different in patient samples of DLBCL, FL, MZL, and MCL compared to normal B cells (9). However, increased levels of *MCL-1* mRNA are suggested to be essential for sustained growth, survival, and resistance to chemotherapeutics in multiple types of lymphoma as well as CLL (54, 55). Unlike BCL-2 and BCL-X, which are overexpressed in a subset of MCL, MCL-1 expression is typically low in this lymphoma (33). Increased levels of *MCL-1* have been observed in multiple myeloma (56) and shown to correlate with disease progression (57).

The expression of Mcl-1 is tightly regulated at both the transcriptional and post-transcriptional level. In contrast to Bcl-2 and Bcl-x, Mcl-1 is a short-lived protein with a half-life of ~30 min compared to the >6 h half-lives observed for Bcl-2 and Bcl-x (124–126). Mcl-1 levels are also regulated by miRNA. Loss of miR-29 and decreased levels of miR-125b and miR-133b, miRNA that bind and negatively regulate *MCL-1* expression (127–130), have been reported in many lymphomas, including Burkitt, anaplastic large cell, and DLBCL, which may also contribute to increased *MCL-1* expression (131–133). Elevated expression of MCL-1 can also be the result of aberrant post-translational mechanisms. For instance, increased expression of the deubiquitinase USP9X, which is responsible for removing polyubiquitin chains that target MCL-1 protein for degradation, correlates with increased MCL-1 protein in FL, DLBCL, and multiple myeloma (134). In addition, increased protein stability of MCL-1 can also lead to protein overexpression as a result of genetic inactivation of FBW7, a ubiquitin ligase (135, 136).

#### A1/BFL-1

Protein-based structural analyses indicate, A1 (BCL2A1), the mouse homolog of human BFL-1, is most highly related to the anti-apoptotic Mcl-1 (137). Because *A1/Bfl-1*-null mice are embryonic lethal, recently, an *A1/Bfl-1*-conditional knockout mouse was generated that was viable, fertile, and only showed minor defects in the hematopoietic system (58, 59). *A1/Bfl-1* transgenic mice demonstrated that overexpression of A1/Bfl-1 did not cooperate with Myc to drive B cell lymphomagenesis (60). However, overexpression of A1/BFL-1 in lymphoma cell lines provided protection from apoptosis induced by a number of stimuli, including ligation of the B cell receptor (138), cytokine deprivation (139), and treatment with staurosporine or etoposide (139, 140). In contrast, knocking down *A1/BFL-1* resulted in increased sensitivity of B cell lymphoma cells to cell death caused by CD20 cross-linking and DNA-damaging drugs (141).

Increased levels of A1/BFL-1 mRNA have been reported in DLBCL and CLL (61–63). However, our analysis of public gene expression data showed that compared to normal B cells, *A1/BFL-1* was not overexpressed in DLBCL, FL, MZL, and MCL (9). We also observed reduced *A1/BFL-1* expression in Burkitt lymphoma patient samples compared to normal human B cells (9). A1/BFL-1 protein can be post-translationally regulated by ubiquitin-mediated proteasomal degradation (139, 140). While regulatory mechanisms, including ubiquitination/proteasomal degradation (139, 140) and direct transcriptional activation by NF-κB (nuclear factor-kappaB) (142) have been reported, the contributions of these mechanisms to increased levels of *A1/BFL-1* in B cell lymphomas have not been evaluated. Based on the available data, the contribution of A1/BFL-1 to B cell lymphomas does not appear to be significant.

#### BCL-W

The gene encoding *BCL-W* (BCL2L2 for Bcl-2 like 2) was initially discovered via a PCR-based strategy while searching for additional *BCL-2* related genes (143). Mice lacking *Bcl-w* were determined to be essentially normal, except for a profound block in male spermatogenesis (64, 65). Several observations have pointed to a potential role for Bcl-w in tumorigenesis. For example, in a mouse model of Myc-driven myeloid leukemogenesis, Bcl-w overexpression cooperated with Myc to accelerate leukemia development (66). In addition, high levels of BCL-W were present in cell lines derived from human lymphomas, leukemias, and multiple solid organ cancers (66). Similar to its anti-apoptotic relatives, overexpression of Bcl-w in mouse B and T lymphocytes imparted resistance to cytotoxic agents (143, 144). Recently, we discovered Bcl-w has a critical, previously unexplored function in B cell survival and lymphomagenesis (8, 9). We demonstrated with mouse models that loss of *Bcl-w* profoundly delayed Myc-induced B cell lymphoma development and sensitized B cells to Myc-induced apoptosis (8). We also evaluated the importance of BCL-W in human lymphomas known to be driven by or reliant on MYC expression, specifically Burkitt and DLBCL. The vast majority of Burkitt lymphoma patient samples examined overexpressed BCL-W at both the mRNA and protein levels (8). When *BCL-W*-specific shRNA was introduced into human Burkitt lymphoma cell lines, they rapidly underwent apoptosis, indicating BCL-W is essential for their continued survival. Additionally, we determined BCL-W was frequently overexpressed in DLBCL. We detected BCL-W mRNA and protein were as often and as highly expressed as BCL-2 in DLBCL, where BCL-2 has long served as the hallmark of a prognostically unfavorable subset (94). Notably, we also observed that increased *BCL-W* mRNA expression correlated with poor patient survival when levels of *BCL-2* mRNA were lower in DLBCL (8).

More recently, we expanded our analyses to explore the additional contributions of BCL-W in other non-Hodgkin B cell lymphomas. We performed an unprecedented analysis of all anti-apoptotic BCL-2 family members across different B cell lymphomas, including Burkitt, DLBCL, FL, MZL, and MCL (9). In all five types of B cell lymphomas, BCL-W was overexpressed compared to normal B cell controls. Increased levels of BCL-W mRNA and protein mirrored those of BCL-2 in FL, which was unexpected, as FL is historically recognized to be driven by a BCL-2 translocation (9, 86–90). Of note, and consistent with previous reports, as the grade of FL increased, levels of *BCL-2* decreased (9, 145), whereas *BCL-W* expression remained elevated in both low and high grade FL (9). Taken together, BCL-W appears to have a critically important, and previously unrecognized, anti-apoptotic role in B cell lymphoma.

**Figure 2:**
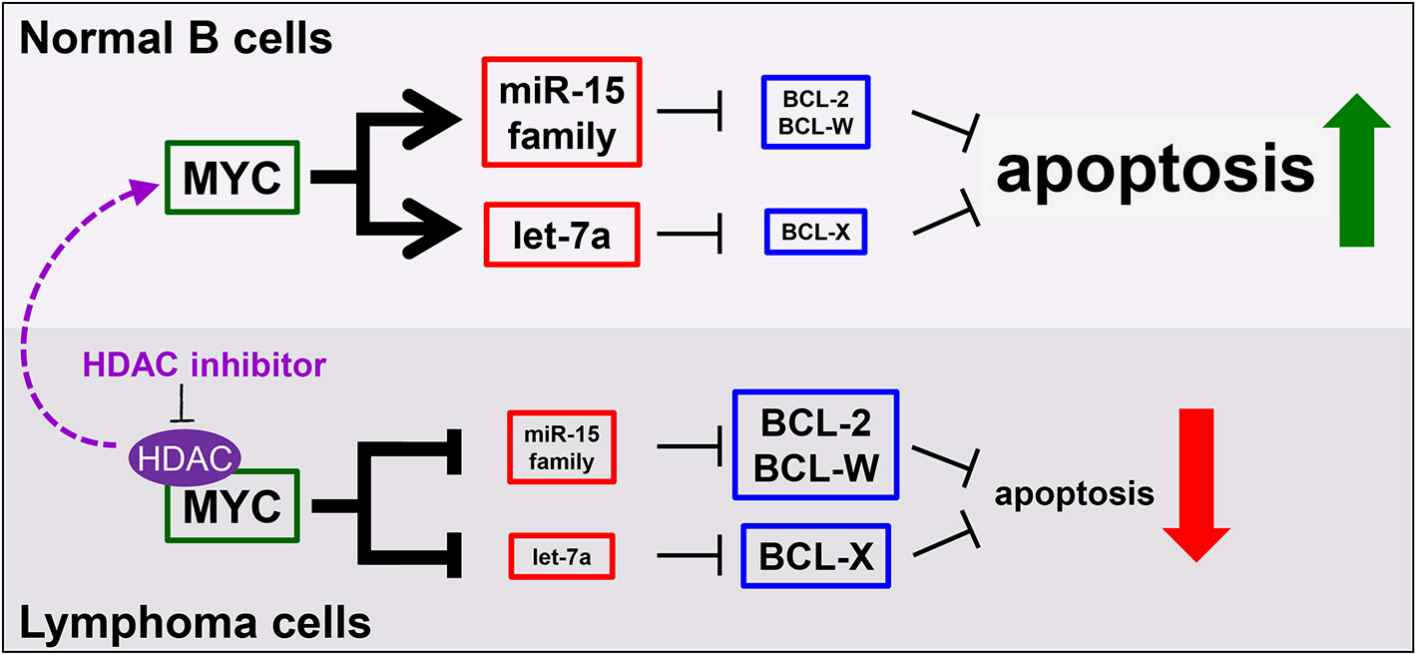
MicroRNA mediate a novel mechanism of Myc-induced apoptosis that is inactivated in malignant cells, but re-activated by HDAC inhibition. In normal B cells **(top)**, MYC transcriptionally upregulates the miR-15 family and let-7a to target and reduce BCL-2, BCL-W, and BCL-X levels, thereby promoting apoptosis. However, in lymphoma cells **(bottom)**, MYC, in complex with histone deacetylases (HDAC), transcriptionally repress the same miRNA, causing increased levels of anti-apoptotic proteins and reduced apoptosis. This mechanism can be re-activated in lymphoma cells by inhibiting HDACs (purple arrow).

Multiple avenues to regulate levels of Bcl-w have been proposed. Overexpression of BCL-W can result from increased activity at the *BCL-W* promoter (146). A number of studies using different tumor types have documented that elevated expression of *BCL-W* may be attributed to the downregulation of miRNA that target *BCL-W* mRNA (128, 147, 148). We recently demonstrated that tumor suppressive miRNA target *BCL-W, BCL-2*, and *BCL-X* as a novel, miRNA-mediated mechanism of apoptosis induced by the oncogenic transcription factor Myc (8, 109, 121). Specifically, in normal cells, Myc transcriptionally activates the miR-15 family and let-7a, which bind and negatively regulate the expression of *BCL-W, BCL-2*, and *BCL-X*, causing cells to undergo apoptosis (Figure 2). However, cancer cells, including lymphoma, inactivate this mechanism. By means that have not been fully elucidated, but do involve histone deacetylase (HDAC) enzymes, Myc switches from a transcriptional activator to a transcriptional repressor of the miR-15 family and let-7a in lymphoma. This, in turn, allows the expression of anti-apoptotic *BCL-W, BCL-2, and BCL-X* to increase, thereby facilitating tumorigenesis (Figure 2). We established a link between regulation of BCL-W expression and Myc. Identifying this novel mechanism of miRNA-mediated apoptosis also answered a question that has remained largely unexplored. Previously, it was shown that Myc suppressed the expression of Bcl-2 and Bcl-x through an unknown, indirect mechanism (149, 150). These recent studies provide direct evidence of a mechanism whereby Myc transcriptionally modulates miRNA expression leading to altered expression of Bcl-w as well as Bcl-2 and Bcl-x (8, 109, 121). Therefore, increased expression of anti-apoptotic Bcl-2 family members, through a variety of mechanisms, are essential survival factors to B cell lymphoma.

## Targeting Anti-Apoptotic Bcl-2 Family Members in Lymphoma

### Biological Rationale, Drug Development, and Early Clinical Studies for Targeting BCL-2

**Figure 3:**
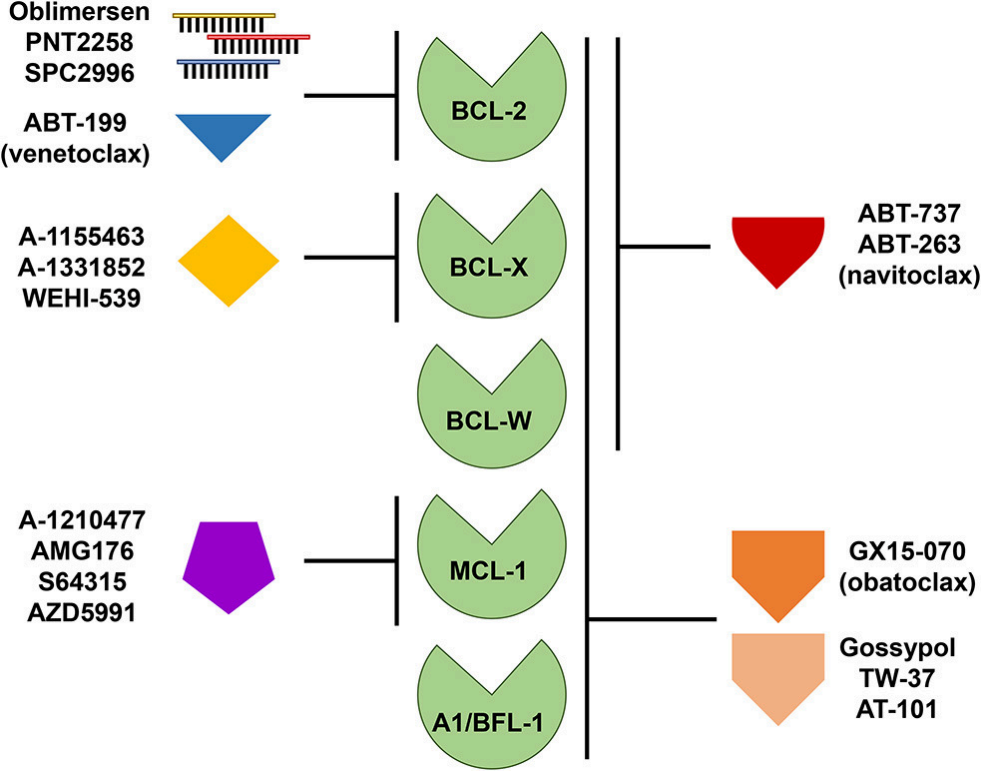
Targeting anti-apoptotic BCL-2 family members for therapy. Therapeutic strategies directed at targeting single or multiple anti-apoptotic BCL-2 family members include the use of antisense oligonucleotides and small molecule inhibitors or mimetics. Like the pro-apoptotic BH-3 only proteins, these small molecules display varying affinities for anti-apoptotic BCL-2 family members as indicated.

Due to the persuasive *in vitro* and *in vivo* evidence that anti-apoptotic BCL-2 family members confer a survival advantage to neoplastic cells, and contribute to chemotherapeutic resistance in different types of hematologic malignancies, including B cell lymphomas, several strategies have been developed to target them. Since BCL-2 was considered the most important anti-apoptotic BCL-2 family member in B cell lymphoma, it was targeted first. Initial attempts to pharmacologically target BCL-2 consisted of decreasing the intracellular levels of BCL-2 with the delivery of RNA antisense molecules (Figure 3). Several of these antisense molecules had encouraging preclinical efficacy and entered clinical trials, including Oblimersen (G3139/Genasense) (151, 152), PNT2258 (NCT02226965) (153, 154), and SPC2996 (NCT00285103) (155). Of the three anti-BCL-2 antisense oligonucleotides, the best characterized was Oblimersen sodium (G3139, Genasense; Genta Inc.). Oblimersen was the first drug to demonstrate proof-of-principle specific down-regulation of BCL-2 protein in human tumors (156, 157) and provided initial preclinical and clinical evidence of synergy with cytotoxic drugs, biological agents, and steroids in a variety of human cancers, including non-Hodgkin lymphoma, multiple myeloma and acute myeloid leukemia (AML) (156–159).

The limited efficacy of Oblimersen in AML (158) and other hematologic malignancies was attributed, in part, to inefficient intracellular delivery and led to the search for small molecules that could target the anti-apoptotic proteins themselves. Each of the anti-apoptotic proteins has a hydrophobic groove where they interact with the BH-3 domains of other BCL-2 family members. With the advances made by structural biology and improved knowledge of the protein-protein interactions within the Bcl-2 family, small molecule inhibitors, known as BH-3 mimetics were developed. BH-3 mimetics, based on their functional mimicry of the BH-3 only pro-apoptotic proteins, can bind with high affinity to the same hydrophobic pocket of one or more anti-apoptotic Bcl-2 family members. The high affinity and specificity of the BH-3 mimetics displaces already-bound or prevents binding of newly synthesized pro-apoptotic BH-3 only proteins from their anti-apoptotic partners, leaving the cells in a “primed” state for apoptosis. This has proven particularly important in combination treatments, where the addition of BH-3 mimetics sensitizes cells to a variety of anti-cancer compounds and aids in circumventing intrinsic or acquired resistance (160).

The first synthetic BH-3 mimetic was ABT-737, a small-molecule that binds with high affinity to BCL-2, BCL-X, and BCL-W, but not MCL-1 or A1/BFL-1 (Figure 3), and was shown to efficiently induce apoptosis at sub-micromolar concentrations in a variety of non-Hodgkin lymphoma cell lines (161, 162) and in primary CLL cells (163, 164). An orally bioavailable derivative of ABT-737, ABT-263 (navitoclax), was later developed. ABT-263 (navitoclax) proved to be efficacious in B cell lymphoma xenograft models as a single agent or in combination with a variety of cytotoxic drugs, including rituximab (165). Of note, ectopic overexpression of BCL-2 in B cell lymphoma cell lines protects from ABT-263 (navitoclax)-induced death *in vitro* (8, 166). We demonstrated that, similar to BCL-2 overexpression, BCL-W overexpression in Burkitt lymphoma cell lines conferred resistance to ABT-737 and ABT-263 (8). Also, ectopic Bcl-w overexpression in primary murine precursor and mature B and T cells induced resistance to ABT-737 (144). In early phase clinical trials, navitoclax showed antitumor activity in indolent B cell malignancies (CLL and FL) (167–169). However, the drug was found to induce rapid and severe, dose-limiting, thrombocytopenia and further studies were stopped. Navitoclax-induced thrombocytopenia is believed to be due to an on-target effect on BCL-X, which is a key factor for platelet survival (165, 170, 171). The hematologic toxicity of navitoclax led to a significant effort to develop a BH-3 mimetic that selectively targeted BCL-2. This effort was successful and produced ABT-199 (venetoclax), an orally bioavailable BCL-2-specific BH-3 mimetic [Figure 3, (172)]. Venetoclax (ABT-199) showed promising efficacy *in vitro* and in xenografts *in vivo* for a variety of hematologic malignancies (e.g., leukemia, CLL, DLBCL) without inducing severe thrombocytopenia (172–174).

Based on these encouraging preclinical data, further clinical development of venetoclax was launched in 2011 (Table 2), with a primary focus on relapsed/refractory (R/R) CLL and NHL (M12-175). There were high expectations of clinical benefit with venetoclax in low grade lymphomas, which historically have served as the canonical disease model for BCL-2 overexpression. The first subjects treated on the M12-175 venetoclax study, at daily doses of 100 or 200 mg, were R/R CLL patients with high nodal and blood tumor burden. These patients experienced very rapid (< 12 h) and dramatic reductions in lymphocyte count and lymph node size, but they also developed severe, life-threatening, tumor lysis syndrome (TLS). In all cases, the TLS was effectively managed, and the patients were able to resume therapy on study. These early data demonstrated the rapid pro-apoptotic effect and therapeutic potential of venetoclax in R/R CLL, but required the development of a mitigating strategy to prevent life-threatening TLS. The strategy included selecting a lower venetoclax starting dose (50 mg) and developing a weekly intra-patient dose ramp-up scheme, in addition to careful prophylaxis and management of TLS. Despite the implementation of this strategy, additional episodes of severe TLS were observed, leading to the death of one patient at the highest dose level (1,200 mg) in a large (*N* = 56) cohort of patients with CLL (175). After the starting dose of venetoclax was further decreased to 20 mg and with even more stringent TLS prophylaxis and monitoring, 60 additional CLL patients were treated in an expansion cohort, with ramp-up dosing up to 400 mg daily. No additional events of severe TLS were observed in this group. The most common Grade 3–4 non-TLS toxicity was Grade 3–4 neutropenia (~40% of patients) (175), which is believed to be an on-target effect of BCL-2 inhibition in neutrophil progenitor cells (180). Other common toxicities included Grade 1–2 gastrointestinal symptoms, such as nausea and diarrhea (~50% or patients). From the standpoint of anti-tumor responses, venetoclax showed highly encouraging efficacy in R/R CLL, with an overall response rate (ORR) of 79%, and a complete response rate (CRR) of 20%, in 116 patients. Most notably, responses were observed in high-risk CLL patients, including those with del(17p). These important early observations in high-risk CLL led to a landmark phase II study where 107 patients with del(17p) CLL were treated with venetoclax with the proven ramp-up dosing to 400 mg daily, with an ORR of 79% and a CRR of 8%. Based on the data from these phase I and II studies, venetoclax received accelerated FDA approval on April 11, 2016 for the treatment of patients with del(17p) CLL who have relapsed after, or are refractory to, ≥1 prior line of therapy. Based primarily on data from 389 R/R CLL patients enrolled in the MURANO clinical trial (NCT02005471), on June 8, 2018 the FDA expanded the approval of venetoclax, in combination with rituximab, to include patients with CLL who progressed after at least one prior therapy, regardless of the presence of del(17p) (181).

**Table 2:** Venetoclax as monotherapy in CLL and B cell lymphoma.

**Disease**	**Phase**	**Enrollment**	**Outcomes (%)**	**Common grade 3–4 AEs (%)**
R/R CLL (175)	I	116	ORR: 79; CR: 20	Neutropenia: 41 Anemia: 12 Thrombocytopenia: 12
CLL (w/ del[17p]) (176)	II	158 (153 were R/R & 5 TN)	ORR: 77; CR: 20	Neutropenia: 40 Anemia: 18 Thrombocytopenia: 15
R/R CLL (w/prior BCRi):	II			
Prior Ibrutinib (177)		91	ORR: 65; CR: 9	Neutropenia: 51 Anemia: 29 Thrombocytopenia: 29
Prior Idelalisib (178)		36	ORR: 67; CR: 8	Neutropenia: 50 Thrombocytopenia: 25 Anemia: 17 Hypokalemia: 11
>1 Prior BCRi (179)		28	ORR: 39; CR: 4	Neutropenia: 43 Anemia: 39 Thrombocytopenia: 25 Hypokalemia: 21 Hypophosphatemia: 21
R/R NHL (7):	I			
Overall MCL FL DLBCL DLBCL-RT WM/MZL		106 28 29 34 7 4/3	ORR: 44; CR: 13 ORR: 75; CR: 21 ORR: 38; CR: 14 ORR: 18; CR: 12 ORR: 43; CR: 0 —	Anemia: 15 Neutropenia: 11

*AE, adverse event; R/R, relapsed/refractory; TN, treatment naïve; ORR, overall response rate; CR, complete response; BCRi, B cell receptor pathway inhibitor; CLL, chronic lymphocytic leukemia; NHL, non-Hodgkin lymphoma; MCL, mantle cell lymphoma; FL, follicular lymphoma; DLBCL, diffuse large B cell lymphoma; RT, Richter transformation; WM, Waldenstrom macroglobulinemia; MZL, marginal zone lymphoma*.

### Clinical Trials With Single Agent Venetoclax in B Cell Lymphomas

In parallel with the clinical development of venetoclax in CLL, the drug's profile was also evaluated in patients with a broad variety of B cell lymphomas, including DLBCL, FL, MCL, lymphoplasmacytic lymphoma/Waldenström macroglobulinemia (LPL/WM), and MZL [Table 2, (7)]. The observed toxicity and the efficacy were significantly different compared to those in CLL. TLS did not occur and severe neutropenia was only observed in 11% of patients. The ORR for the entire cohort was 44%, with the best responses seen in MCL (75% ORR; 21% CRR). Remarkably, despite the well-characterized overexpression of BCL-2 in FL, ORR in FL was only 38%. A slightly higher ORR of 44% was noted in patients with FL treated at ≥1,200 mg compared with 27% in those treated at ≤ 900 mg, suggesting that higher doses could lead to better efficacy in the nodal disease, which is the primary disease site in FL. Results in DLBCL were even more disappointing, with ORR of 18% and no clear association between BCL-2 protein expression levels and response.

### Combination Treatments With Venetoclax in Clinical Trials With CLL and B Cell Lymphoma

In light of the low single agent activity of venetoclax in B cell lymphomas, combination regimens are now being evaluated, mostly in the context of the general strategy of adding venetoclax to well-recognized chemo-immunotherapy combination with established efficacy (bendamustine and rituximab; CHOP and rituximab; CHOP and obinutuzumab; EPOCH and rituximab), or highly promising new drugs (ibrutinib) (Tables 3, 4). A phase II trial comparing a venetoclax/rituximab doublet to the three-drug combination of venetoclax/bendamustine/rituximab is ongoing in patients with R/R FL (NCT02187861), with preliminary evidence of superior efficacy with the three drug combination vs. the doublet (ORR 64 vs. 33%) (194). A combination of venetoclax with obinutuzumab in previously untreated FL is also currently underway (NCT02877550). As in CLL, the combination of ibrutinib and venetoclax is being evaluated in a phase II study in relapsed/refractory MCL (NCT02471391). The early experience in this trial suggests good tolerability and promising efficacy of the combination (196). There are also ongoing studies exploring whether venetoclax may act as a chemosensitizing agent, such as a study of venetoclax administered in combination with R-CHOP or obinutuzumab-CHOP in previously untreated DLBCL (NCT02055820). A preliminary report indicated that, as expected, CR rates were high, but follow-up was too short to assess progression-free survival (PFS). The toxicity profile was acceptable, but venetoclax dosing had to be reduced to administration only on days 1 to 10 (rather than continuous dosing) to mitigate the rate of Grade 3–4 neutropenia. A phase II study of venetoclax and dose-adjusted R-EPOCH in patients with Richter transformation to DLBCL has begun (NCT03054896), in addition to a study using a similar regimen for patients with *de novo* aggressive B cell lymphomas, including double-hit DLBCL (NCT03036904). A phase I study is also ongoing to examine the combination of venetoclax and the SYK tyrosine kinase inhibitor TAK-659 for patients with R/R DLBCL and FL (NCT03357627).

**Table 3:** Venetoclax in combination with anti-neoplastic agents in CLL and B cell lymphoma.

**Intervention/Disease**	**Phase**	**Enrollment**	**Outcomes (%)**	**Common grade 3–4 AEs (%)**
R + V in R/R CLL (182)	Ib	49	ORR: 86; CR: 51	Neutropenia: 53 Thrombocytopenia: 16 Anemia: 14 Febrile Neutropenia: 12 Leukopenia: 12 Pyrexia: 12
R + V vs. R + B in R/R CLL (183)	III	R + V: 194	24-mo PFS est: 84.9; ORR: 93.3; CR: 26.8	Neutropenia: 57.7 Anemia: 10.8 Thrombocytopenia: 5.7 Febrile Neutropenia: 3.6
		R + B: 195	24-mo PFS est: 36.3; ORR: 67.7; CR: 8.2	Neutropenia: 38.8 Anemia: 13.8 Thrombocytopenia: 10.1 Febrile Neutropenia: 9.6
I + V in R/R CLL (184)	II	38	ORR: 100; CR: 47	Neutropenia: 19/25 pts 76 Infection: 5/25 pts 20
I + V in R/R and TN high risk CLL (185)	II	R/R: 29	ORR: 100; CR: 64 (14 evaluable pts)	Atrial Fibrillation: 11
		TN: 32	ORR: 100; CR 56 (16 evaluable pts)	
G + V in TN CLL in pts with coexisting medical conditions (186)	III	12 (from run-in phase)	ORR: 100; CR: 58	Neutropenia: 58.3 Febrile Neutropenia: 25 Thrombocytopenia: 16.7 Infection: 16.7 Laboratory TLS: 16.7 Syncope: 16.7
V + BR vs. V + BG in TN or R/R CLL (187)	Ib	55	
V + BR in R/R		30	ORR: 96; CR: 26 (27 evaluable pts)	Neutropenia: 63 Thrombocytopenia: 27 Infection: 27 Anemia: 20 Diarrhea: 10
V + BR in TN		17	ORR: 100; CR: 43 (14 evaluable pts)	Neutropenia: 71 Thrombocytopenia: 24 Anemia: 29 Febrile Neutropenia: 12
V + BG in TN		8	ORR: 100; CR: 43 (7 evaluable pts)	Thrombocytopenia: 63 Neutropenia: 25 Fatigue: 13 Infusion reaction: 13
V + G in TN CLL (188)^*^	Ib	32	ORR: 100; CR: 56.3	Neutropenia: 40.6 Febrile Neutropenia: 12.5 Thrombocytopenia: 12.5
V + G in R/R CLL (189)^*^	Ib	26	ORR: 100; CR: 23.5 (17 evaluable pts)	Neutropenia: 47 Infection: 19 Laboratory TLS: 13
G + I + V in R/R CLL (190)	Ib	12	ORR: 92; CR: 42 (6 evaluable pts)	Neutropenia: 33 Lymphopenia: 17 Thrombocytopenia: 17 Hypertension: 25 Fatigue: 17
G + I + V in TN CLL (191)	II	25	ORR: 100; CR: 50 (23 evaluable pts)	Neutropenia: 44 Leukopenia: 36 Thrombocytopenia: 36 Lymphopenia: 32 Hypertension: 20
B (debulking) -> G + V in TN and R/R CLL (192)	II	35 TN	ORR: 100; CR: 50 (34 evaluable pts)	Neutropenia: 44 Thrombocytopenia: 12 Infection: 14
		31 R/R	ORR: 90; CR: 28 (29 evaluable pts)	
V + R-CHOP vs. V + G-CHOP in TN (91%) and R/R NHL (193):	I	56 (FL 24; DLBCL 17; MZL 5; Composite 5; Other 5)		Neutropenia: 46 Febrile Neutropenia: 29 Thrombocytopenia: 21
V + R-CHOP		21	ORR: 85.7; CR: 67 (21 evaluable pts)	
V + G-CHOP		21	ORR: 81; CR: 62 (21 evaluable pts)	
V + BR vs. BR vs. V + R in RR FL (194):	II			
V + BR		51	ORR: 68; CR: 50 (22 evaluable pts)	Neutropenia: 59 Thrombocytopenia: 39 Febrile Neutropenia: 10
BR		51	ORR: 64; CR: 41 (22 evaluable pts)	Neutropenia: 24 Thrombocytopenia: 6
V + R		53	ORR: 33; CR: 14 (52 evaluable pts)	Neutropenia: 27 Thrombocytopenia: 8
I + V in R/R or TN MCL (195)		24 (23 were R/R)	ORR: 71; CR: 63 (At week 16)	Neutropenia: 25

*AE, adverse event; R/R, relapsed/refractory; TN, treatment naïve; ORR, overall response rate; CR, complete response (includes complete remission with incomplete hematologic recovery); PFS, progression free survival; BCRi, B cell receptor pathway inhibitor; CLL, chronic lymphocytic leukemia; MCL, mantle cell lymphoma; FL, follicular lymphoma; DLBCL, diffuse large B cell lymphoma; RT, Richter transformation; MZL, marginal zone lymphoma; NHL, non-Hodgkin lymphoma; TLS, tumor lysis syndrome; pts, patients*.

*Treatment Abbreviations: V, Venetoclax; R, Rituximab; B, Bendamustine; I, Ibrutinib; G, Obinutuzumab*.

* *Data were obtained from same trial, but 2017 ASH abstract only contained updated data on TN patients. Abstract from 2015 contained data on R/R (14) and TN (6) patients*.

**Table 4:** Ongoing venetoclax combination studies in B cell lymphomas and CLL.

	**Phase**	***N***	**Population**	**Trial number**
**B CELL LYMPHOMAS**				
Ven + Ibrutinib	I	28	MCL R/R	NCT02419560
Ven + Ibrutinib	II	24	MCL first line	NCT02471391
Ven + Ibrutinib + Obinu	I/II	48	MCL R/R	NCT02558816
Ven + Benda + Ritux (BR) vs. BR	II	164	FL R/R	NCT02187861
Ven + Obinu	I	25	FL first line	NCT02877550
Ven + CHOP + Ritux or Obinu	I/II	248	1L DLBCL	NCT02055820
Ritux-DA-EPOCH	II	20	Richter's	NCT03054896
**CLL**				
Ven + Rituximab	Ib	49	R/R	NCT01682616
Ven + Benda + Ritux or Obinu	Ib	100	First line + R/R	NCT01671904
Ven + Obinutuzumab	Ib	81	First line + R/R	NCT01685892
Ven + Benda followed by Obinu	II	66	First line + R/R	NCT02401503
Ven + Rituximab	III	391	R/R	NCT02005471
Ven + Obinu	III	445	First line	NCT02242942
Ven + Ibrutinib	II	78	First line + R/R	NCT02756897
Ven + Ibrutinib, Obinu	II	40	First line del(17p)	NCT02758665
Ven + Ibrutinib, Obinu	I/II	68	First line + R/R	NCT02427451

*N, number enrolled; Ven, Venetoclax; Benda (or B), Bendamustine; Ritux (or R), Rituximab; Obinu, Obinutuzumab; MCL, mantle cell lymphoma; FL, follicular lymphoma; DLBCL, diffuse large B cell lymphoma; R/R, relapsed/refractory; CLL, chronic lymphocytic leukemia*.

### Inhibiting Other Anti-apoptotic BCL-2 Family Members

The accompanying toxicities that come with targeting multiple BCL-2 family members have fueled the development of new inhibitors to selectively target specific anti-apoptotic BCL-2 family members. Furthermore, individual lymphomas may be differentially reliant on one or more of the anti-apoptotic BCL-2 family members, which can be exploited therapeutically. In addition to the BCL-2 specific inhibitors developed, BCL-X and MCL-1 inhibitors have also been generated (5). There are currently no known inhibitors specific for BCL-W or A1/BFL-1.

#### BCL-X Inhibitors

While compounds selective for BCL-2 over BCL-X have shown anti-tumor effects *in vivo* with limited platelet toxicities (175, 180), not all cancers express BCL-2 or require BCL-2 for their continued survival (5, 197). The first reported BCL-X selective inhibitor with sub-nanomolar affinity and selectivity was WEHI-539, which antagonized the anti-apoptotic activity of BCL-X (198). However, WEHI-539 has been limited to *in vitro* studies as a tool compound due to its unfavorable chemical properties for use *in vivo* (198). Other efforts using high-throughput screening with nuclear magnetic resonance and structure-based medicinal chemistry led to the development of the BCL-X selective inhibitors A-1155463 and its orally bioavailable analog A-1331852 [Figure 3, (199, 200)]. Cell lines from a variety of malignancies, including AML showed sensitivity to the BCL-X inhibitors. *In vivo* studies in mice indicated inhibition of BCL-X with A-1331852 as a single agent reduced the volume of a T-cell leukemia, whereas venetoclax did not (180). In addition, the mice tolerated the BCL-X inhibitor. These data suggest that some cancers appear to be dependent on BCL-X and not on BCL-2 and that targeting one over the other may be beneficial.

#### MCL-1 Inhibitors

Multiple approaches have been explored to selectively target MCL-1. Initially, stabilized alpha-helices of BH-3 only proteins [known as “stapled” peptides (201)] and alpha- or beta-peptide foldamers (202) were designed to target MCL-1. Several compounds with affinity for MCL-1 were identified by screening libraries of natural compounds and small molecules. However, many of these have reported off-target or minimal effects (203, 204). Although obatoclax (GX15-070) targets other anti-apoptotic BCL-2 family members, it was one of the first BH-3 mimetics reported to inhibit MCL-1 [Figure 3, (205)]. Obatoclax has completed phase I/II clinical trials for a number of malignancies, including CLL, but with modest efficacy and neutropenia as a common toxicity (206). At this time, no further clinical trials with obatoclax are ongoing. Similar to obatoclax, TW-37, a gossypol derivative, has shown some toxicity, but has demonstrated potency as a single agent in the treatment of DLBCL cell lines and synergized with other chemotherapies in xenograft models [Figure 3, (207)]. Phase I clinical trials for AT-101, a more refined variant of the gossypol family of pan-BCL-2 family inhibitors, show that it is well-tolerated with treatable neutropenia (208). Two clinical trials of AT-101 as a single agent in R/R B cell lymphomas (NCT00275431) and in combination with rituximab in CLL (NCT00286780) have been completed, but final results have not been published.

A-1210477 was the first selective MCL-1 inhibitor to demonstrate picomolar binding to MCL-1 while maintaining selectivity for MCL-1 over other anti-apoptotic family members [Figure 3, (209)]. A-1210477 forms complexes with MCL-1, thereby disrupting endogenous MCL-1:BIM complexes. As a single agent, this compound induced apoptosis in MCL-1-dependent multiple myeloma cell lines (210). However, A-1210477 does not demonstrate the necessary pharmacokinetics for *in vivo* use. AMG176 was the first putative MCL-1 inhibitor to undergo clinical evaluation (NCT02675452) (211), but no data in humans has been reported. In addition, phase I trials are actively recruiting or soon to recruit patients for testing the MCL-1 specific inhibitors S64315 (MIK665) in patients with R/R DLBCL and multiple myeloma, AML, and myelodysplastic syndrome (NCT02992483, NCT02979366, and NCT03672695) and AZD5991 in patients with R/R hematologic malignancies, CLL, T-cell lymphoma, and multiple myeloma (NCT03218683). A question that remains largely unanswered is whether normal cells will tolerate MCL-1 inhibition at the level necessary for therapeutic benefit. Potential on-target toxicities may include cardiac (212, 213), hepatic (214, 215), and hematological (45–47), which are based on those observed in *Mcl-1* knockout mouse models (45–47, 212–215).

### Combination Treatments With Other Anti-apoptotic BCL-2 Family Inhibitors

At present, there are a limited number of clinical trials dedicated to evaluating the effects of anti-apoptotic BCL-2 family inhibitors aside from venetoclax either alone or in combination with other therapies. Navitoclax, which inhibits BCL-2, BCL-X, and BCL-W, was being tested in combination with bendamustine and rituximab in patients with relapsed DLBCL as part of the NAVIGATE study, but recruitment was terminated due to non-safety-related reasons (NCT01423539). A study of navitoclax together with venetoclax and chemotherapy (including peg-asparaginase, vincristine, dexamethasone, and tyrosine kinase inhibitor) is ongoing for patients with R/R ALL or R/R lymphoblastic lymphoma (NCT03181126). A phase I study evaluating the safety of navitoclax administered in combination with rituximab is active for patients with CD20-positive lymphoproliferative disorders and CLL (NCT00788684). In addition, obatoclax mesylate (GX15-070MS), which inhibits BCL-2, BCL-X, BCL-W, and MCL-1 (Figure 3) has been evaluated as a single agent followed by a combination with rituximab for treatment naive patients with FL (NCT00427856). Obatoclax has also been combined with bortezomib for the treatment of R/R MCL (NCT00407303). A phase I/II trial was initiated to study the side effects and the dose of obatoclax when given together with rituximab and bendamustine in treating patients with R/R non-Hodgkin lymphoma including MZL, FL, and MCL; however, the study was withdrawn due to lack of patient accrual (NCT01238146). A phase I dose escalation study of the MCL-1 selective inhibitor S64315 in combination with venetoclax (estimated start date: December 3, 2018) will be testing the safety and tolerability in patients with AML (NCT03672695). The continued efforts to develop novel anti-apoptotic BCL-2 family protein inhibitors will continue to pave the way for new clinical trials combining current inhibitors with both conventional and other novel agents in various lymphomas.

### Resistance Mechanisms for BCL-2 Family Protein Inhibitors

Given that venetoclax selectively inhibits BCL-2, this compound should be effective in cancer cells that express BCL-2; however, this does not always occur. For example, FL expresses high levels of BCL-2 due to its *t*(14;18) translocation; yet, the clinical response rate to venetoclax is quite low in FL patients (7). This suggests BCL-2 expression alone is insufficient to predict BCL-2 dependence. In a recent study, a subset of lymphoma cell lines expressing BCL-2 protein were resistant to venetoclax, resulting from acquired mutations in BCL-2 and the pro-apoptotic protein BAX or a phosphorylation event on BCL-2 that prevented venetoclax from binding, thereby blocking apoptosis (216–218). Similarly, upon comparing venetoclax-resistant FL cells with their parental cell lines, the resistant FL cells had significantly higher levels of ERK1/2 and BIM phosphorylation at serine 69, targeting BIM for proteasomal degradation; thus, reducing the pro-apoptotic nature of the cells (219, 220). Another study showed increased phospho-ATK levels in a venetoclax-resistant B cell lymphoma line, suggesting activation of the PI3K pathway (221). Whole-exome sequencing and methylation profiling of serial CLL samples from eight patients before venetoclax treatment and at the time of venetoclax resistance did not show genetic alterations in *BCL-*2 (222). However, most patients acquired mutations in cancer-related genes, including *BRAF, NOTCH1, RB1*, and *TP53*, and had homozygous deletion of *CDKN2A/B*.

Resistance of FL and DLBCL to single agent venetoclax may be attributed, in part, to elevated levels of other anti-apoptotic family members. For example, we recently reported that BCL-W was elevated in both FL and DLBCL at a similar frequency as BCL-2 (9). Moreover, our recent large-scale evaluation of all anti-apoptotic BCL-2 family members in multiple non-Hodgkin lymphomas revealed that many selected for the overexpression of more than one anti-apoptotic family member, simultaneously (9). These data provide a potential explanation into why just targeting BCL-2 with venetoclax did not result in high complete response rates for FL or DLBCL (7). Moreover, levels of BCL-X and MCL-1 were upregulated in venetoclax-resistant DLBCL cell lines (221). Furthermore, in lymphoma cell lines that have become resistant to navitoclax, increased levels of anti-apoptotic A1/BFL-1 and MCL-1 are observed (223).

With the increasing focus on MCL-1 as an important regulator of apoptosis in leukemia and lymphoma (224) and potential mediator of venetoclax resistance, a number of efforts are in progress to better define the role of MCL-1 in venetoclax resistance and to develop strategies to downregulate MCL-1 levels as a possible way to overcome it. While MCL-1 specific inhibitors are in early phase of development (225, 226), several studies have already shown that downregulation of MCL-1 mRNA and/or protein levels in BH-3 mimetic-resistant cells increases the sensitivity to navitoclax and venetoclax (54, 227). For example, the pan CDK inhibitor dinaciclib restored sensitivity to navitoclax- and venetoclax-mediated apoptosis in resistant lymphoma cells via inhibition of MCL-1 phosphorylation by CDK2/Cyclin E, which in turn led to the destabilization of MCL-1 protein and release of BIM from MCL-1 (228). The combination of dinaciclib and venetoclax resulted in robust synergistic cell death in DLBCL cell lines and in primary CLL patient samples. Additional ongoing strategies to enhance the therapeutic efficacy of venetoclax in B cell neoplasms (increasing response rate, depth of response, overcoming primary and secondary resistance), include combinations with BTK inhibitors (229), dual delta- and gamma-PI3 kinase inhibitors (230), SYK inhibitors (231), and BET inhibitors (232). While all these studies are of very high interest, and many of the preclinical concepts are being evaluated in clinical trials, there is a need to better understand the specific mechanistic functions of the BCL-2 family members in each of these pro-apoptotic combinations, where they are redundant and where they may lead to synthetic lethality.

With clinical trials on-going and many more being developed using anti-apoptotic BCL-2 family inhibitors for treatment of B cell lymphoma, as well as other hematologic malignancies and solid-organ cancers, there remains a significant lack of knowledge of these proteins and their requirements in non-Hodgkin lymphomas. Aside from FL and DLBCL, alterations in specific anti-apoptotic BCL-2 family members have not been well-characterized or associated with other B cell lymphomas, which is likely due to the lack of a comprehensive analysis prior to the one we recently published (9). Therefore, it is likely necessary to measure and monitor the levels of BCL-2 family members when enrolling patients onto clinical trials testing selective anti-apoptotic inhibitors. Inhibiting one anti-apoptotic BCL-2 family member that is not expressed in that lymphoma should have no effect on the lymphoma and be unnecessary treatment for that patient. Additionally, targeting one anti-apoptotic BCL-2 family member may lead to the dependency on another, ultimately leading to therapeutic resistance.

### Non-apoptotic Effects of BCL-2 Family Inhibition

There have been reports suggesting that inhibition of anti-apoptotic BCL-2 family members may activate cell signaling pathways, in addition to apoptosis, to further promote survival and resistance to cell death. For example, a recent proteomic analysis following treatment of DLBCL and MCL cell lines with venetoclax showed venetoclax treatment (both short- and long-term) altered the levels of proteins involved in apoptosis, but also the expression levels and phosphorylation status of proteins involved in the DNA damage response (i.e., γH2AX, CHK2, ATM), growth/survival signaling pathways (i.e., PTEN, Src, MAPK, AKT), and cellular metabolism (i.e., HK2, PDK1, PKM2, GCLM) were also affected (233). However, further studies are necessary to determine whether these effects are directly attributable to venetoclax-mediated BCL-2 inhibition and the resulting apoptosis or effects that are independent of BCL-2. The BCL-2 family of proteins has also been reported to function in maintaining calcium homeostasis (234). The early BH-3-only protein mimetic HA14-1 was shown to dysregulate intracellular calcium signaling in platelets, reportedly due to off-target effects, but neither navitoclax nor venetoclax disrupted intracellular calcium transport mechanisms (235, 236). Furthermore, investigations have shown that BCL-2 family proteins may be involved in autophagy (237). Specifically, studies have reported that navitoclax and the BH-3-mimetic HA14-1 can block the interaction between BCL-2/BCL-X and Beclin1, a protein important for the localization of autophagic proteins, leading to the activation of Beclin1-dependent autophagy (237–240). BCL-2 family BH-3-only mimetics can also regulate cell autophagy through activation of the unfolded protein response signaling pathway PERK-eIF2α-ATF4, which upregulates expression of the autophagy gene Atg12 (241–243).

## Conclusion

Defects in apoptosis are universal to all B cell lymphomas and defects in apoptotic signaling is frequently associated with resistance to chemotherapy (2, 4, 244). In this review, we have highlighted the Bcl-2 family network of proteins in lymphoma, including the recent discovery of BCL-W overexpression in B cell lymphomas, and described current clinical strategies to inhibit anti-apoptotic Bcl-2 family proteins that aim to develop more effective therapies for B cell lymphoma. Despite decades of significant progress in identifying the molecular underpinnings of apoptotic cell death and their contributions to the pathogenesis, survival, and resistance to treatment of individual B cell lymphomas, recent efforts have revealed that several critical factors have been significantly underestimated. The identification of BCL-W as a previously unrecognized key contributor to B cell lymphoma has substantially aided in increasing our understanding of the BCL-2 family and the alterations in their expression that contribute to B cell lymphoma survival and therapy resistance. This new knowledge has opened the door to the development of additional selective cancer therapeutics and combination therapies that may redefine the treatment of B cell lymphoma. Revealing the involvement of BCL-W in many types of B cell lymphoma has also opened the door to studying its possible role in treatment resistance. To fully exploit the potential of selective inhibitors of anti-apoptotic BCL-2 proteins for the treatment of B cell lymphoma, we must know which inhibitors should be given to which patients. To guide the use of specific inhibitors in individual patients, or molecularly defined patient subsets, we must know which anti-apoptotic BCL-2 protein(s) are the most relevant target(s). For example, while one malignancy may have requirements for BCL-2, another might require BCL-X and/or BCL-W, highlighting the importance of using the right biomarkers to evaluate each lymphoma. In the era of personalized medicine, these recent advances attest to the power of discoveries in basic science being directly translated into the clinic to improve targeted treatment strategies for individual lymphomas.

## Author Contributions

CA, SC-G, PP, and CE contributed to the literature review, and to the writing and editing of the material presented in this manuscript.

### Conflict of Interest Statement

The authors declare that the research was conducted in the absence of any commercial or financial relationships that could be construed as a potential conflict of interest.
